# Associations of first-trimester TMAO and its precursors with gestational diabetes mellitus: a pilot prospective cohort study

**DOI:** 10.3389/fnut.2025.1587863

**Published:** 2025-06-16

**Authors:** Geng-Dong Chen, Ting-Ting Pang, Peng-Sheng Li, Shao-Xin Ye, Xiao-Yan Gou, Hai-Yan Wang, Dong-Xin Lin, Da-Zhi Fan, Lu-Sha Deng, Li-Juan Wang, Zi-Xing Zhou

**Affiliations:** ^1^Foshan Institute of Fetal Medicine, Foshan Women and Children Hospital Affiliated to Guangdong Medical University, Foshan, Guangdong, China; ^2^Foshan Key Laboratory of Stem Cell and Maternal-Child Health, Foshan Women and Children Hospital, Foshan, China; ^3^Stem Cell Clinical Research Center, Foshan Women and Children Hospital, Foshan, China; ^4^Department of Medical Records, Foshan Women and Children Hospital Affiliated to Guangdong Medical University, Foshan, Guangdong, China; ^5^Department of Obstetrics, Foshan Women and Children Hospital Affiliated to Guangdong Medical University, Foshan, Guangdong, China

**Keywords:** trimethylamine N-oxide (TMAO), TMAO precursors, L-carnitine, gestational diabetes mellitus, pregnant women

## Abstract

**Aims:**

We aimed to provide a comprehensive understanding of the associations between Trimethylamine-N-oxide (TMAO), its precursor and gestational diabetes mellitus (GDM).

**Methods:**

In this prospective study, 940 women were included in a Chinese single -center pregnant cohort. First trimester plasma concentrations of TMAO and its precursors (betaine, choline, L-carnitine, and trimethylamine) were measured using UPLC-ESI-MS/MS. GDM and specific abnormal glucose levels (fasting glucose; one-hour post-load glucose, 1-h PG; two-hour post load glucose, 2-h PG; and 1-h PG ≥ 8.6 mmol/L) were assessed through oral glucose tolerance tests. First-trimester plasma concentrations of TMAO and its precursors were divided into quartile groups (bottom, Q1; middle, Q2 and Q3; top, Q4).

**Results:**

Among the subjects, 167 (17.8%) were found to have GDM. After adjusting for potential covariates, the lower groups (Q1) of L-carnitine were associated with a higher risk of GDM compared to the reference group (middle quartiles). The OR (95% CI, p) was1.56 (1.04, 2.35, *p* = 0.032) for L-carnitine. Specifically, the associations were mainly derived from L-carnitine and abnormal 1-h PG. The ORs (95% CI, p) were 2.00 (1.24, 3.24, *p* = 0.005).

**Conclusion:**

Low plasma levels (bottom vs. middle quartiles) of L-carnitine the first-trimester pregnancy were associated with a higher risk of GDM and abnormal 1-h PG in Chinese pregnant women.

## Background

Globally, gestational diabetes mellitus (GDM) has become a major public health issue, leading to a higher risk of maternal and infant perinatal adverse events (1), as well as an increased long-term risk of type 2 diabetes and adverse outcomes for offspring (2, 3). In 2021, according to the International Association of Diabetes in Pregnancy Study Group (IADPSG)’s criteria, the prevalence of GDM is approximately 14% globally, with 27.6% in Middle East and North Africa (highest), 20.8% in South-East Asia, and 7.1% in North America and Caribbean (lowest) (4). A system review reported that the prevalence of GDM in mainland China from 2010 to 2017, based on 25 studies and 79,064 subjects, was 14.8% (15.7% in the north and 20.3% in the south) (5). There is a need to develop more strategies for the early prevention of GDM and to curb the rapid growth of the condition.

Trimethylamine-N-oxide (TMAO) can be directly supplemented from dietary fish intake and also endogenously derived from its precursors, including betaine, choline, and L-carnitine. These precursors are converted into trimethylamine (TMA) by gut microbiota and further oxidized to form TMAO in the liver. Betaine and choline can be obtained from dietary egg intake, while L-carnitine is primarily sourced from red meat consumption (6–9).

Trimethylamine-N-oxide was reported to increase the risk of cardiovascular disease (CVD) and the occurrence of type 2 diabetes mellitus (T2DM) (10, 11), as well as their adverse consequences such as mortality (12). The influence of TMAO precursors on T2DM may vary, with higher choline levels increasing the risk of T2DM (13), while higher betaine and L-carnitine levels are associated with a reduced T2DM risk (13, 14). TMAO and its precursors have also been studied during pregnancy and are suggested to be related to several pregnancy complications, such as missed abortion and preeclampsia (15, 16). Regarding GDM, several studies have been conducted, revealing inconsistent relationships between TMAO and its precursors and GDM (16–25). A double-design study (comprising one case–control study, with cases/controls set at 433/433, and one nested case–control study, with cases/controls set at 276/552) reported higher plasma TMAO levels during 24–32 gestational weeks or <20 gestational weeks to be associated with an increased risk of GDM (24). In contrast, a separate nested case–control study (cases/controls: 243/243) revealed that early pregnancy TMA was associated with an elevated GDM risk, while TMAO was associated with a reduced GDM risk (23). Additionally, GDM patients in Greece were observed to have lower TMAO levels (20). Besides, a number of articles have reported no significant associations between TMAO and GDM (18, 21, 22). In a prospective study of 368 Canadian women, TMAO levels during pregnancy were not associated with GDM, while GDM was associated with increased plasma TMAO levels in cord blood (25). Furthermore, an association was identified between higher maternal blood levels of TMAO (1–3 days after delivery) and higher odds of GDM among 1,496 US women (16). Similar inconsistent associations with GDM were also identified for TMAO precursors, such as choline, which has been found to have detrimental (17, 18), protective (16, 20, 23), and null associations (19, 21, 22); betaine [protective (18, 19, 22, 23, 25), and null associations (16, 17, 20, 21)]; and L-carnitine [detrimental (18), protective (17, 18, 23), and null associations (16, 21)].

The heterogeneity of these results may be attributable to the varying gestational weeks of TMAO testing during pregnancy, with many studies conducting tests during the second trimester, concurrently or shortly before the OGTT test. Previous studies indicated that the status of GDM may also influence TMAO levels (16, 25). An MR-based study also suggests that TMAO and carnitine do not increase the risk of T2DM, whereas T2DM increases TMAO levels, and possible causal inversion may exist in some studies (26). Therefore, in order to better investigate the relationships of TMAO and its precursors on the development of GDM, investigation in the first trimester can reduce the possibility of causal inversion. The extant research on TMAO and GDM in early pregnancy has yielded scant evidence and provided little information on specific glucose levels at different times of the OGTT. Further research should be conducted in order to provide more evidence of earlier pregnancy TMAO and its precursors in relation to GDM.

Recently, the IDF released a statement suggesting that 1-h post-load plasma glucose (1-h PG) may have advantages over 2-h post-load plasma glucose (2-h PG) or fasting glucose for the diagnosis of intermediate hyperglycaemia (27). During the OGTT tests, glucose levels at 0, 1, or 2 h might reflect different degrees or types of glucometabolic impairment (28). Therefore, subdividing specific abnormal glucose levels from the OGTT may be more informative and insightful compared to a single outcome of GDM. The IDF also suggested 1-h PG ≥ 8.6 mmol/L as a diagnostic criterion for intermediate hyperglycemia (27). Whether imbalanced TMAO metabolism is associated with specific abnormal glucose outcomes (abnormal fasting glucose, 1-h PG, 2-h PG, and 1-h PG ≥ 8.6 mmol/L) during pregnancy is unclear and needs further investigation.

In this prospective study, we aimed to investigate the relationships between first trimester plasma concentrations of TMAO and several of its precursors (betaine, choline, L-carnitine, and trimethylamine, TMA) with the outcomes of GMD and specific abnormal glucose levels (abnormal fasting glucose, 1-h PG, 2-h PG, and 1-h PG ≥ 8.6 mmol/L) in 940 Chinese pregnant women.

## Methods

### Subjects

This study is based on a preliminary analysis of a prospective cohort conducted at Foshan Women and Children Hospital in Foshan City, Guangdong Province, China (29, 30). Subjects were recruited from August 14, 2019, to January 31, 2021. Inclusion criteria included women aged ≥ 18 years, diagnosed as pregnant in the first trimester (up to 14 weeks’ gestation), and willing to deliver their babies. Subjects with the following conditions were excluded: cardiovascular disease, diabetes, cancer, chronic kidney disease, and mental disorders. A total of 987 subjects donated blood samples, and their plasma concentrations of trimethylamine N-oxide (TMAO) and its precursor were measured. Subsequently, 47 subjects were excluded for the following reasons: (1) twins or multiple pregnancies; (2) blood samples collected after 14 weeks of gestation; (3) missing core data. The final study population comprised 940 pregnant women. The study was conducted in accordance with the Declaration of Helsinki and was approved by the Ethics Committee of Foshan Women and Children Hospital (FSFY-MEC-2019-025). Written informed consent was obtained from all subjects.

### Measurement of TMAO and its precursors

Baseline plasma samples in the first trimester were donated during the initial antenatal visit. The samples were kept at 4°C, separated within 6 h of collection, and stored at −80°C until analysis. Trimethylamine N-oxide (TMAO) and five of its precursors, including betaine, choline, L-carnitine, and TMA, were measured using UPLC-ESI-MS/MS, which was performed by Shanghai Luming Biotechnology Co., Ltd. The coefficient of variation (CV) values of these indicators ranged from 4.75 to 9.46% among 7 mixed plasma quality control samples. Detailed information on the measurement of TMAO and its precursors is provided below.

Sample pre-processing: take 100 μL of the sample and add 300 μL of a methanol-acetonitrile (2:1, v/v) mixture (containing 0.01 mol/L BHT and the isotopic internal standard L-carnitine-D3); vortex for 30 s, then perform ultrasonic extraction in an ice water bath for 5 min; leave at −20°C for 30 min. Centrifuge for 15 min (4°C, 13,000 rpm), take 200 μL of the supernatant, add 300 μL of a methanol–water (2:98, v/v) mixture, vortex for 30 s, and perform ultrasonic extraction in an ice-water bath for 3 min. Add 300 μL of chloroform, vortex for 30 s, and leave at 4°C for 10 min. Centrifuge for 5 min (4°C, 13,000 rpm), then transfer 150 μL of the supernatant to a brown LC injection bottle and store at −80°C until analysis. Quality control (QC) samples were prepared by mixing the extracts from all samples in equal volumes.

The UPLC-ESI-MS/MS analytical method was used to qualitatively and quantitatively detect the target metabolites, and the specific analytical conditions and methods were as follows: Chromatographic conditions: Injection volume: 5 μL; Mobile phase: A (0.1% formic acid-water solution), B (methanol); Gradient Elution Procedures (GEP): 0 min A/B (99:1, V/V), 1.6 min A/B (99:1, V/V), 2.2 min A/B (2:98, V/V), 5 min A/B (2:98, V/V), 5.01 min A/B (99:1, V/V), 6 min A/B (99:1, V/V). Mass spectrometry conditions: Curtain gas: 35 psi; Collision-activated dissociation (CAD) parameters: medium; Positive ion spray voltage: 5500 V; Ion source temperature: 600°C; Gas 1: 60 psi; Gas 2: 50 psi.

### Measurement of GDM

An oral glucose tolerance test (OGTT) during 24–28 gestational weeks was performed for pregnant women in this study. Specific abnormal blood glucose was defined if they met the following criteria of OGTT tests: fasting blood glucose ≥5.1 mmol/L; 1-h post-load glucose (1-h PG) ≥ 10.0 mmol/L; or 2-h post-load glucose (2-h PG) ≥ 8.5 mmol/L. Information on abnormal fasting glucose, 1-h PG, and 2-h PG was then collected. GDM was diagnosed in pregnant women who met any of the criteria for abnormal glucose mentioned above. We further investigated another outcome of 1-h PG ≥ 8.6 mmol/L, which represents intermediate hyperglycemia as suggested in a recent article (27).

### Potential covariates

Subjects participated in face-to-face interviews, during which a structured questionnaire was used to collect baseline demographic and socioeconomic data (29). Potential covariates in this study included age, education status (senior high school or below, junior college, bachelor’s degree or above), history of parity and gravidity, and family income (less than or equal to 5,000, 5,000–10,000, or greater than 10,000 yuan per month). Maternal pre-pregnancy weight 3 months before pregnancy was self-reported. Baseline maternal height was measured with subjects wearing light clothing and no shoes, and the measurements were accurate to 0.1 cm. Pre-pregnancy body mass index (BMI) was calculated. Gestational weight gain was determined by subtracting pre-pregnancy weight from the weight measured at the OGTT appointment.

### Statistical analysis

Continuous variables were represented by Mean ± Standard deviation or median (interquartile range), and tested by student-t tests or Mann–Whitney *U* test. Categorical variables were represented by frequency (percentage) and tested by Chi-square test. The plasma concentrations of TMAO and its precursors were divided into quartile groups, with the bottom quartile (Q1) representing the lowest concentration and the top quartile (Q4) representing the highest concentration. Middle quartiles (Q2 and Q3) were designated as the reference. Differences in TMAO and its precursor concentrations among the various quartile groups were tested using the Kruskal–Wallis test. Logistic regression analyses were used to explore associations between TMAO and its precursors and the risk of GDM and specific abnormal glucose levels. Two adjustment models were used. Model 1 wan unadjusted (univariate analysis), and Model 2 was adjusted for age, pre-pregnancy BMI, parity, gravidity, education, family income, and gestational weight gain (GWG). Restricted cubic spline (RCS) regressions (with Model 2 adjustment) using three knots (at the 10th, 50th, and 90th quantiles) were employed to investigate the dose–response relationships between TMAO, its precursors, and the risk of GDM and specific abnormal glucose levels. Likelihood ratio tests were used for assessing non-linearity in these relationships. The statistical analyses were conducted using SPSS 21.0 for Windows (SPSS, Inc., Chicago, United States), and R statistics software version 4.3.1. RCS and forest plots were created using R 4.3.1 software. A two-tailed *p*-value of 0.05 was considered statistically significant.

## Results

In this study, a total of 940 pregnant women were included. The participants had a mean age of 30.1 ± 4.4 years, a mean pre-pregnancy BMI of 20.5 ± 2.7 kg/m^2^, and a mean GWG of 7.0 ± 3.3 kg. Among the subjects, 167 (17.8%) were found to have GDM, and the mean glucose levels were 4.4 ± 0.4 mmol/L for fasting glucose, 7.8 ± 1.8 mmol/L for 1-h PG, and 6.8 ± 1.4 mmol/L for 2-h PG. The measurement of TMAO and its precursors was performed on blood samples collected in the first trimester (mean: 10.7 ± 1.8 gestational weeks). Detailed information can be found in Table 1. Subjects with GDM (vs. Non-GDM) tended to be older, have a higher pre-pregnancy BMI, more gravidity times, and elevated levels of fasting glucose, 1-h PG, 2-h PG, and TMA concentrations, along with lower betaine concentrations (*p* < 0.05).

**Table 1:** Characteristic of subjects.

Variables	Total subjects	Non-GDM	GDM	*p*-value
*N*, (%)	940	773 (82.2)	167 (17.8)	
Age, years	30.1 ± 4.4	29.8 ± 4.3	31.9 ± 4.4	**<0.001**
Pre-pregnancy BMI, kg/m^2^	20.5 ± 2.7	20.3 ± 2.6	21.6 ± 3.2	**<0.001**
Gestational weight gain, kg	7.0 ± 3.3	6.9 ± 3.2	7.3 ± 3.7	0.152
Gravidity, times	2.1 ± 1.2	2.1 ± 1.2	2.3 ± 1.4	**0.034**
Parity, times	1.5 ± 0.6	1.5 ± 0.6	1.6 ± 0.7	0.054
Maternal education				0.426
Senior high school or below, *N* (%)	375 (39.9)	301 (38.9)	74 (44.3)	
Junior college, *N* (%)	310 (33.0)	260 (33.6)	50 (29.9)	
Bachelor or above, *N* (%)	255 (27.1)	212 (27.4)	43 (25.7)	
Family income, Yuan per month				0.516
≤5,000, *N* (%)	365 (38.8)	303 (39.2)	62 (37.1)	
5,000–10,000, *N* (%)	407 (43.3)	337 (43.6)	70 (41.9)	
>10,000, *N* (%)	168 (17.9)	133 (17.2)	35 (21.0)	
Fasting glucose, mmol/L	4.4 ± 0.4	4.4 ± 0.3	4.8 ± 0.5	**<0.001**
One-hour post-load glucose, mmol/L	7.8 ± 1.8	7.3 ± 1.4	10.1 ± 1.4	**<0.001**
Two-hour post-load glucose, mmol/L	6.8 ± 1.4	6.4 ± 1.0	8.6 ± 1.6	**<0.001**
Gestational weeks for measuring TMAO and its precursors, weeks	10.7 ± 1.8	10.6 ± 1.7	10.9 ± 2.3	0.061
Plasma concentrations of TMAO-related indicators				
Betaine, ng/mL^a^	3,642 (2,983, 4,436)	3,691 (2,993, 4,466)	3,423 (2,903, 4,223)	**0.038**
Choline, ng/mL^a^	1,157 (833, 1,477)	1,146 (831, 1,459)	1,184 (848, 1,622)	0.111
L-Carnitine, ng/mL^a^	13,480 (11,851, 14,774)	13,482 (11,934, 14,784)	13,468 (11,429, 14,640)	0.243
TMAO, ng/mL^a^	10.9 (6.5, 17.4)	10.8 (6.6, 17.5)	11.2 (6.3, 16.8)	0.763
TMA, ng/mL^a^	19.9 (16.0, 25.3)	19.7 (15.9, 24.9)	21.1 (16.8, 27.6)	**0.047**

^a^Median (IQR) of the plasma concentrations of TMAO-related indicators.

BMI, body mass index; GDM, gestational diabetes mellitus; TMAO, trimethylamine-N-oxide; TMA, trimethylamine. Bold font represents *p* < 0.05.

The plasma concentrations of TMAO and its precursors were divided into quartile groups: bottom quartile (Q1, lowest), middle quartiles (Q2 and Q3), and top quartile (Q4). The distribution (median and interquartile range) of the concentrations of TMAO and its precursors among these groups is presented in Table 2. Significant differences in TMAO and its precursor concentrations were observed among the different quartile groups (all *p* < 0.001).

**Table 2:** Distribution of plasma concentrations of TMAO-related indicators.

TMAO precursors	Plasma concentrations of TMAO-related indicators						*p*-value
	Bottom quartile (Q1)		Middle quartiles (Q2 and Q3)		Top quartile (Q4)		
	Median (IQR)	Range	Median (IQR)	Range	Median (IQR)	Range	
	*N* = 235		*N* = 470		*N* = 235		
Betaine, ng/mL	2,645 (2,383, 2,852)	(1,337, 2,983)	3,642 (3,292, 3,989)	(2,984, 4435.7)	5,088 (4,740, 5,806)	(4,436, 10,287)	**<0.001**
Choline, ng/mL	599 (444, 731)	(57.1, 832.77)	1,157 (989, 1,311)	(832.8, 1476.8)	1793 (1,592, 2085)	(1476.9, 3,659)	**<0.001**
L-Carnitine, ng/mL	10,068 (7,942, 11,290)	(8,652, 11,841)	13,480 (12,736, 14,126)	(11,851, 14,759)	15,654 (15,164, 16,542)	(14,774, 19,347)	**<0.001**
TMAO, ng/mL	4.3 (2.9, 5.4)	(0, 6.53)	10.9 (8.7, 13.5)	(6.54, 17.36)	24.2 (19.9, 33.5)	(17.41, 523.6)	**<0.001**
TMA, ng/mL	13.5 (12.0, 14.9)	(7.3, 15.995)	19.9 (18.1, 21.9)	(16.00, 25.26)	30.7 (27.5, 37.0)	(25.27, 72.92)	**<0.001**

^a^*p*-value was tested by Kruskal–Wallis test.

TMAO, trimethylamine-N-oxide; TMA, trimethylamine. Median (IQR) of the plasma concentrations of TMAO-related indicators.

Bold font represents *p* < 0.05.

**Figure 1:**
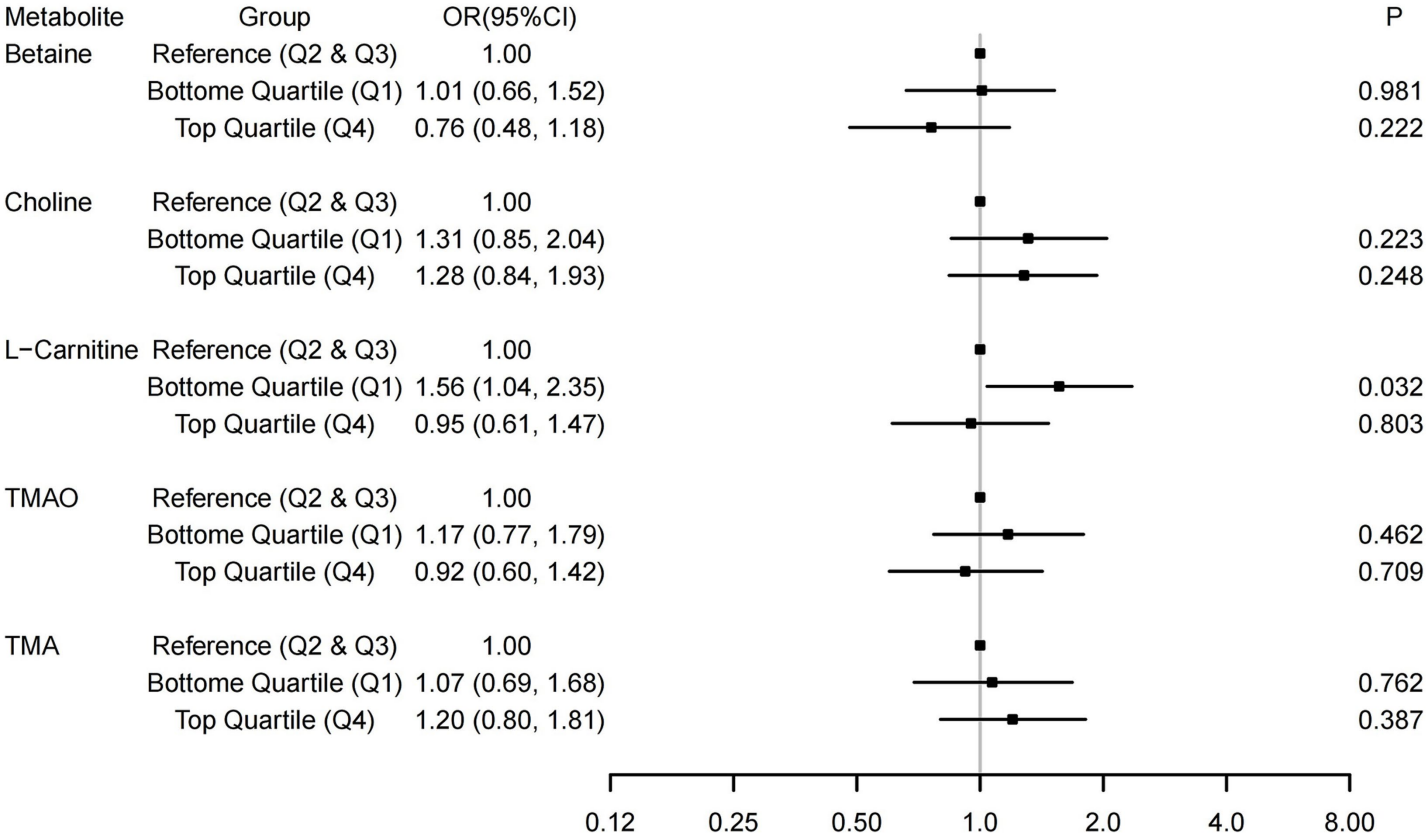
Associations between TMAO and its precursors and the risk of Gestational diabetes mellitus (GDM). The associations were adjusted for covariates in Model 2, which included age, pre-pregnancy BMI, parity, gravidity, education, family income, and gestational weight gain.

Logistic regression analyses were used to investigate the associations of TMAO and its precursors with the risk of GDM, with the middle quartile groups treated as the reference. As shown in Supplementary Table 1, in the univariate analysis (Model 1), the bottom quartile groups of L-carnitine were associated with a higher risk of GDM. After adjusting for several potential covariates (Figure 1), compared with the reference (middle quartiles), the lower groups of L-carnitine remained associated with a higher risk of GDM. The OR (95% CI, p) was 1.56 (1.04, 2.35, *p* = 0.032) for L-carnitine. In sensitivity analyses (Supplementary Table 2), the results tended to be more pronounced among subjects aged <30 years and those with a pre-pregnancy BMI < 20.13 kg/m^2^, but no significant interactions were found (*P*-interaction = 0.196 and 0.442). For every standard deviation increase in L-carnitine concentration, the risk of abnormal 1-h PG decrease is reduced (OR: 0.78, 95% CI: 0.63, 0.95, *p* = 0.013). No significant associations were observed between other TMAO-related indicators (per standard deviation increase) and GDM or specific abnormal glucose levels (Supplementary Table 3).

The associations of TMAO and its precursors with the risk of specific abnormal glucose levels (abnormal fasting glucose, 1-h PG, 2-h PG, and 1-h PG ≥ 8.6 mmol/L), adjusted for covariates in Model 2, are presented in Supplementary Figures 1–4. Compared to the reference, low levels (bottom quartile group) of L-carnitine were associated with a higher risk of abnormal 1-h PG. The OR (95% CI, p) was 2.00 (1.24, 3.24, *p* = 0.005) for L-carnitine. Both low (bottom quartile) and high (top quartile) levels of choline were marginally (but did not reach significance) associated with the risk of abnormal 2-h PG. The ORs (95% CI, p) were 1.67 (0.99, 2.97, *p* = 0.055) and 1.64 (1.00, 2.70, *p* = 0.052), respectively. No significant associations were found between the bottom or top quartile of the exposure and the outcomes of abnormal fasting glucose and 1-h PG ≥ 8.6 mmol/L.

**Figure 2:**
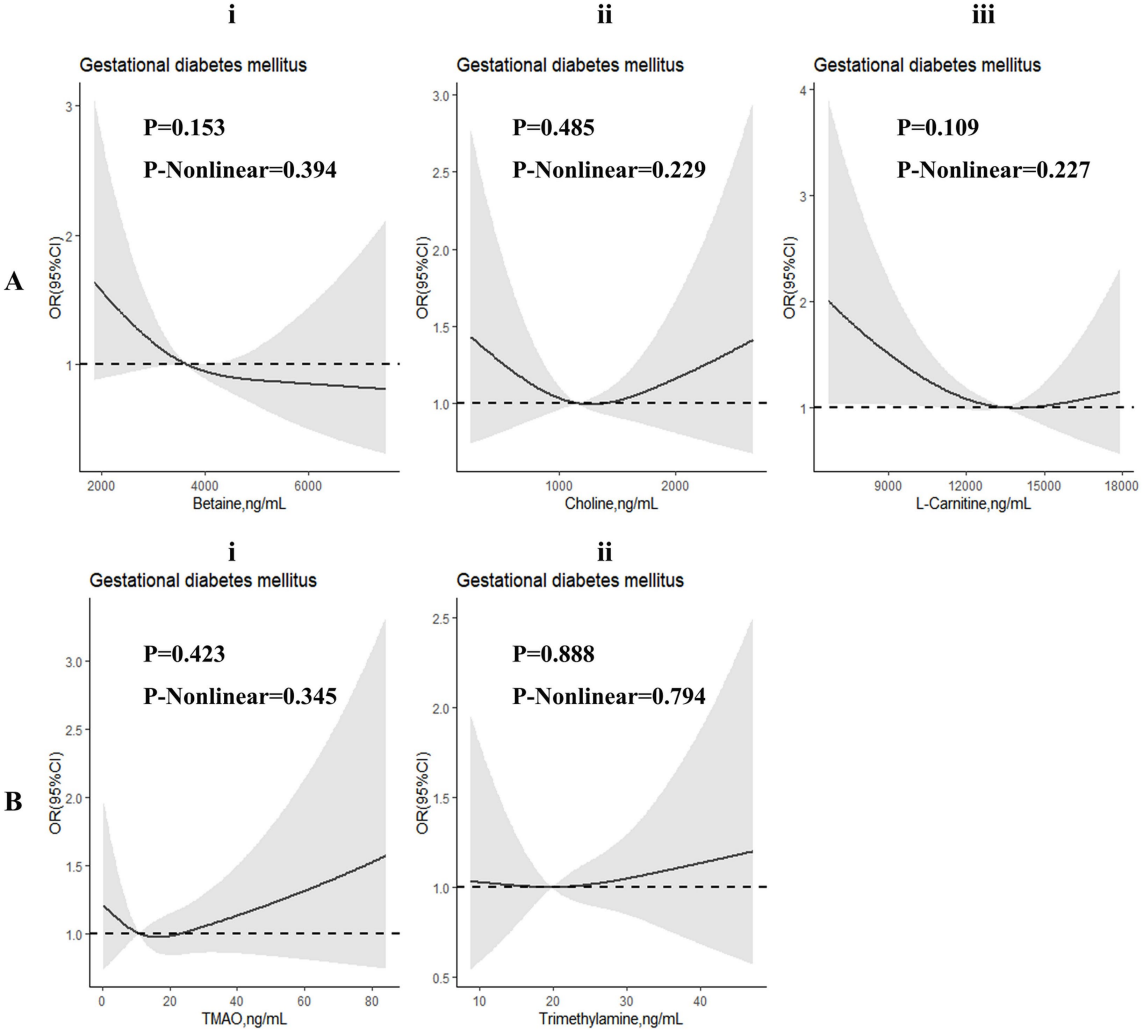
Dose-response associations between TMAO and its precursors and gestational diabetes mellitus (GDM). Analyses were performed by restricted cubic spine regressions (3 knots). Parts **(Ai–iii,Bi,ii)** represent the dose-response associations of betaine, choline, L-carnitine, trimethylamine N-oxide (TMAO) and trimethylamine (TMA), respectively.

Restricted cubic spline regressions were performed to investigate dose–response associations between TMAO and its precursors and the risk of GDM (Figure 2) and abnormal glucose levels (Supplementary Figure 5). Although we found that the associations in many RCS images tended to be L-shaped or U-shaped, no significant non-linear associations were identified in these images.

## Discussion

In this prospective study of 940 Chinese pregnant women, compared to the reference group (middle quartile, Q2 and Q3), we found that low plasma levels (bottom quartile, Q1) of L-carnitine were associated with a higher risk of GDM and abnormal 1-h PG in Chinese pregnant women.

### Main findings on TMAO and GDM associations

Our study revealed no significant association between first-trimester TMAO levels and GDM risk. These findings align with previous studies reporting neutral roles of TMAO across investigations (16–18, 21, 22, 25), while it has also been variably characterized as detrimental (24) or protective (20, 23) in other studies. The heterogeneity in reported outcomes may stem from differences in gestational timing of TMAO measurement. Existing evidence suggests potential bidirectional causality, as GDM may elevate TMAO levels during mid-to-late pregnancy (18, 21, 22, 24, 25) and postpartum periods (16, 25). Notably, first-trimester TMAO investigations remain scarce. While Huo et al. (23) reported a protective association between early-pregnancy TMAO and GDM, this finding lacks robust replication. In this study, we prospectively investigated the associations between first-trimester TMAO levels and GDM as well as specific abnormal glucose levels in a relatively large sample, which avoids possible causal inversions. Although no significant results were found for TMAO and GDM, our contribution adds to the limited evidence base in this domain; however, though definitive conclusions require further large-scale prospective studies.

### TMAO precursors and GDM risk

We identified an inverse relationship between low L-carnitine levels and increased GDM risk, consistent with select prior investigations (17, 23). Higher (vs. lower) levels of L-carnitine were associated with a 70.7% lower GDM risk (OR: 0.293, 95CI: 0.134, 0.638) in a case–control study (cases/controls:201/201) (17). Lower L-carnitine levels (≤112 vs. >112 nmol/mL) in early pregnancy were associated with a higher risk of GDM (OR: 13.5, 95CI: 5.50, 33.2) in a nested case–control study (cases/controls:243/243) (23). However, two null-association studies warrant consideration: one employed a cross-sectional design with limited sample size (22), while the other measured TMAO postpartum (16). A contradictory study reported reduced GDM risk with low L-carnitine, but this analysis utilized blood samples collected during oral glucose tolerance test (OGTT) administration (18)—a methodological limitation given potential confounding by acute metabolic changes. The heterogeneity of these results may partly stem from the fact that TMAO precursors are measured at different gestational stages. The prospective design and the use of first-trimester data in our study can both avoid causal inversion and provide research evidence for early prevention and intervention of GDM during the first trimester.

Neither betaine nor choline demonstrated statistically significant associations with GDM in our cohort. Null associations of betaine (16, 17, 20, 21) or choline (19, 21, 22) with GDM were also found in several previous studies, which were inconsistent with several other studies. For instance, choline has been paradoxically linked to both increased (17, 18) and decreased (16, 20, 23) GDM risk across studies, while betaine generally shows protective trends (18, 19, 22, 23, 25). These discrepancies may reflect gestational timing variations in metabolite measurement or reference group selection biases. Nevertheless, our study provides a comprehensive picture of the association between a range of TMAO precursors and GDM, as well as specific abnormal glucose levels in the first trimester, in a prospective study with a large sample size. In particular, the association of TMAO precursors with 1-h PG ≥ 8.6 mmol/L (as suggested by IDF) has not been reported before. These results will provide reference evidence for subsequent studies. Concentrations of TMAO and its precursors may vary as pregnancy advances. From 12 to 16 weeks of gestation to the time of delivery, plasma free choline and TMAO levels rose by 49 and 13%, while betaine levels decreased by 21% (31). It was suggested that L-carnitine levels also decrease during pregnancy (32, 33). Although there was no statistical association of choline, TMAO, and betaine with GDM in our study, the fluctuating levels of these biomarkers during pregnancy may weaken the association of the bottom quartile group of choline and TMAO with GDM and strengthen the association of the top quartile group as pregnancy progresses. In contrast, for betaine and L-carnitine, decreasing levels during pregnancy may have strengthened the association of the bottom quartile group with GDM while weakening the association of the top quartile group as pregnancy advances. More studies, especially with TMAO precursors measured in different trimesters, are still needed to further illustrate the problems.

### Mechanistic considerations for L-carnitine

The observed association between low L-carnitine and elevated GDM/abnormal 1-h plasma glucose (1-h PG) risk finds mechanistic support in clinical trials. A meta-analysis of 21 RCTs (2,041 T2DM patients) demonstrated that 1 g/day L-carnitine supplementation improves fasting glucose, HbA1c, BMI, and triglyceride levels (34). Pooled analysis of 41 RCTs (2,900 adults) revealed 12-week L-carnitine intervention significantly enhances insulin sensitivity and reduces HOMA-IR/HbA1c (35). An RCT reported that a 12-week L-carnitine supplement (2,970 mg/d) was associated with improved insulin sensitivity and lower fasting plasma glucose levels in T2DM patients (36). These evidences may suggest that moderate L-carnitine may reduce the risk of GDM by regulating glucose levels and lipid metabolism in the body. Several other potential mechanisms may help further explain these results. L-carnitine may improve insulin resistance by inducing autophagy through PPARγ and removing dysfunctional mitochondria in the skeletal muscle of a high-fat-diet-induced rodent model of obesity (37). L-carnitine transports long-chain fatty acids across the inner mitochondrial membrane and improves the beta-oxidation of long-chain fatty acyl Coenzyme A (CoA), the accumulation of which leads to insulin resistance (38, 39). Additionally, L-carnitine may enhance fatty acid oxidation through the activation of AMPK/PGC1α signaling both *in vivo* and *in vitro*, helping to alleviate obesity-related adverse symptoms (40). Finally, L-carnitine is involved in forming an effective transport system for acetyl or acyl groups out of the mitochondria, improving metabolic inflexibility and insulin sensitivity by regulating the mitochondrial acetyl-CoA/CoA ratio and acyl-CoA/CoA ratio (38, 41).

### Clinical implications

Red meat—a primary L-carnitine source—is associated with elevated T2DM and CVD risks (42). Additionally, the clinical implications of L-carnitine related to GDM risk are still limited. Therefore, our findings caution against increasing L-carnitine levels through supplementation or increased red meat consumption. Dietary L-carnitine derives from multiple sources including poultry, fish, eggs, and dairy products. Our results suggest maintaining adequate (but not excessive) L-carnitine levels may mitigate GDM risk, whereas deficiency potentiates risk. This aligns with our previous work linking low iron status (another red meat-associated nutrient) to GDM susceptibility (29). We observed no GDM risk reduction with elevated L-carnitine levels, emphasizing the need for balanced and healthy nutritional approaches to keep L-carnitine within a suitable range rather than in an extreme state through an unhealthy diet or unguided supplementation.

### Strength and limitations

This study has strengths in its prospective design, with exposure (TMAO and its precursors) measured in the first trimester and outcomes measured by OGTT during 24 to 28 gestational weeks, which avoids possible causal inversions. Additionally, a series of TMAO and its precursors (betaine, choline, L-carnitine, and trimethylamine) and the outcomes of specific abnormal blood glucose levels (fasting glucose; 1-h PG; 2-h PG; and 1-h PG ≥ 8.6 mmol/L) were investigated, which may provide a more comprehensive understanding of the associations between exposure and outcomes.

Our study had the following limitations: Firstly, although it was conducted in the largest obstetric center in Foshan City, which serves a large population, subjects from a single center may introduce geographical and demographic biases. Thus, the sample’s homogeneity and generalizability are limited, and future multi-center cohorts are needed to validate our results. Secondly, although we measured a series of plasma concentrations of TMAO and its precursors, the measurements were performed only once in the first trimester, making it impossible to assess their dynamic changes throughout the entire pregnancy. Therefore, the temporal trends and time-dependent associations between TMAO and its precursors with GDM could not be investigated in this study. Multiple assessments over the course of pregnancy would likely provide more comprehensive data, which should be explored in the future. Thirdly, with the absence of dietary data and fecal samples for the detection of gut microbiota composition, we are unable to adjust for these potential covariates or explore the interactions of these factors in our study. More studies are encouraged to include these factors to provide more comprehensive results in this field. Finally, insulin levels were not assessed in this study; therefore, we could not investigate whether the associations of L-carnitine and GDM were due to an improvement in insulin sensitivity, which remains to be addressed by further high-quality studies.

## Conclusion

In this prospective study, we found that low plasma levels (bottom vs. middle quartiles) of L-carnitine were associated with a higher risk of GDM and abnormal 1-h PG in Chinese pregnant women. Although several marginally significant relationships were observed, no significant associations were found for TMAO or its other precursors with GDM and specific abnormal blood glucose. More high-quality studies with larger sample sizes are needed for further examination of our results. Our study further emphasizes the importance of maintaining balanced blood concentrations of L-carnitine during early pregnancy for the prevention of GDM and specific abnormal blood glucose levels.

## Data Availability

The raw data supporting the conclusions of this article will be made available by the authors, without undue reservation.
