# Supplementary material for: Associations of first-trimester TMAO and its precursors with gestational diabetes mellitus: a pilot prospective cohort study
Source: Front Nutr. 2025 Jun 16;12:1587863. doi: 10.3389/fnut.2025.1587863 (PMC12206624; doi:10.3389/fnut.2025.1587863)
Supplement: Supplementary file 1 [file Table_1.docx]

**Associations of First-Trimester TMAO and Its Precursors with Gestational Diabetes Mellitus: A Pilot Prospective Cohort Study**

Geng-dong Chen^1#*^, Ting-ting Pang^#3^, Peng-sheng Li^1^, Shao-xin Ye^1^, Xiao-yan Gou^1^, Hai-yan Wang^1^, Dong-xin Lin^1^, Da-zhi Fan^1^, Lu-sha Deng^3^**^*^**, Li-juan Wang^2^**^*^**, Zi-xing Zhou**^*^**^1^,

^1^ Foshan Institute of Fetal Medicine, Foshan Women and Children Hospital Affiliated to Guangdong Medical University, Foshan city, Guangdong Province, 528000, China;

^2^ Department of Obstetrics, Foshan Women and Children Hospital Affiliated to Guangdong Medical University, Foshan city, Guangdong Province, 528000, China;

^3^ Department of Medical Records, Foshan Women and Children Hospital Affiliated to Guangdong Medical University, Foshan city, Guangdong Province, 528000, China;

**Supplemental Table-1** Associations of TMAO-related indicators with gestational diabetes mellitus (univariate model)

|  | Middle quartiles (Q2 and Q3) | Bottom quartile (Q1) | | | Top Quartile (Q4) | | |
| --- | --- | --- | --- | --- | --- | --- | --- |
|  | *Reference* | *OR* | *95%CI* | *p* | *OR* | *95%CI* | *p* |
| Betaine, ng/mL | 1.00 | 1.08 | (0.72, 1.61) | 0.728 | 0.76 | (0.49, 1.17) | 0.211 |
| Choline, ng/mL | 1.00 | 1.27 | (0.82, 1.95) | 0.285 | 1.39 | (0.93, 2.08) | 0.107 |
| L-Carnitine, ng/mL | 1.00 | **1.53** | **(1.02, 2.27)** | **0.038** | 0.95 | (0.62, 1.47) | 0.823 |
| TMAO, ng/mL | 1.00 | 1.13 | (0.75, 1.7) | 0.569 | 0.98 | (0.65, 1.49) | 0.93 |
| TMA, ng/mL | 1.00 | 1.03 | (0.66, 1.59) | 0.912 | 1.3 | (0.87, 1.94) | 0.198 |

Bold font means statistically significant.

**Supplemental Table-2** Associations of L-Carnitine with gestational diabetes mellitus (sensitivity analysis)

|  | GMD | | | | | | |  |
| --- | --- | --- | --- | --- | --- | --- | --- | --- |
|  | Reference | Bottom Quartile(Q1) | | | Top Quartile (Q4) | | |  |
| L-Carnitine |  | OR | 95% (CI) | P | OR | 95% (CI) | P | P-interaction |
| Age<Median | 1.00 | **2.54** | **(1.25, 5.18)** | **0.010** | 1.32 | (0.60, 2.94) | 0.49 | 0.196 |
| Age≥Median | 1.00 | 1.20 | (0.73, 1.98) | 0.478 | 0.81 | (0.47, 1.38) | 0.431 |  |
|  |  |  |  |  |  |  |  |  |
| Pre-pregnancy BMI<Median | 1.00 | **1.90** | **(1.00, 3.60)** | **0.049** | 0.74 | (0.34, 1.62) | 0.45 | 0.442 |
| Pre-pregnancy BMI≥Median | 1.00 | 1.37 | (0.80, 2.34) | 0.250 | 0.98 | (0.56, 1.71) | 0.942 |  |

Median for age was 30 years, and for pre-pregnancy BMI was 20.13 kg/m^2^.

The associations were adjusted for covariates in Model 2, which included age, pre-pregnancy BMI, parity, gravidity, education, family income, and gestational weight gain.

**Supplemental Table-3** Associations of TMAO-related indicators (per one standard deviation increase) with gestational diabetes mellitus.

|  | GDM | | | Abnormal fasting glucose | | | Abnormal 1-h PG | | | Abnormal 2-h PG | | | Abnormal 1-h PG≥8.6 mmol/L | | |
| --- | --- | --- | --- | --- | --- | --- | --- | --- | --- | --- | --- | --- | --- | --- | --- |
|  | *OR* | *95%CI* | *p* | *OR* | *95%CI* | *p* | *OR* | *95%CI* | *p* | *OR* | *95%CI* | *p* | *OR* | *95%CI* | *p* |
| Betaine | 0.86 | (0.71, 1.03) | 0.101 | 0.85 | (0.62, 1.17) | 0.328 | 0.82 | (0.66, 1.03) | 0.089 | 0.90 | (0.72, 1.12) | 0.336 | 0.91 | (0.79, 1.06) | 0.210 |
| Choline | 1.02 | (0.85, 1.21) | 0.85 | 0.88 | (0.65, 1.20) | 0.410 | 0.98 | (0.79, 1.21) | 0.847 | 1.04 | (0.85, 1.28) | 0.716 | 1.06 | (0.92, 1.23) | 0.409 |
| L-Carnitine | 0.87 | (0.74, 1.03) | 0.108 | 0.84 | (0.63, 1.11) | 0.222 | **0.78** | **(0.63, 0.95)** | **0.013** | 0.86 | (0.71, 1.05) | 0.149 | 0.95 | (0.82, 1.10) | 0.473 |
| TMAO | 1.10 | (0.92, 1.32) | 0.287 | 0.96 | (0.73, 1.27) | 0.767 | 1.11 | (0.94, 1.31) | 0.226 | 1.13 | (0.94, 1.36) | 0.179 | 1.06 | (0.91, 1.23) | 0.473 |
| TMA | 1.05 | (0.89, 1.25) | 0.552 | 0.99 | (0.74, 1.32) | 0.923 | 1.02 | (0.83, 1.26) | 0.831 | 1.09 | (0.89, 1.33) | 0.389 | 1.05 | (0.91, 1.21) | 0.541 |

The associations were adjusted for covariates in Model 2, which included age, pre-pregnancy BMI, parity, gravidity, education, family income, and gestational weight gain.

Bold font means statistically significant.


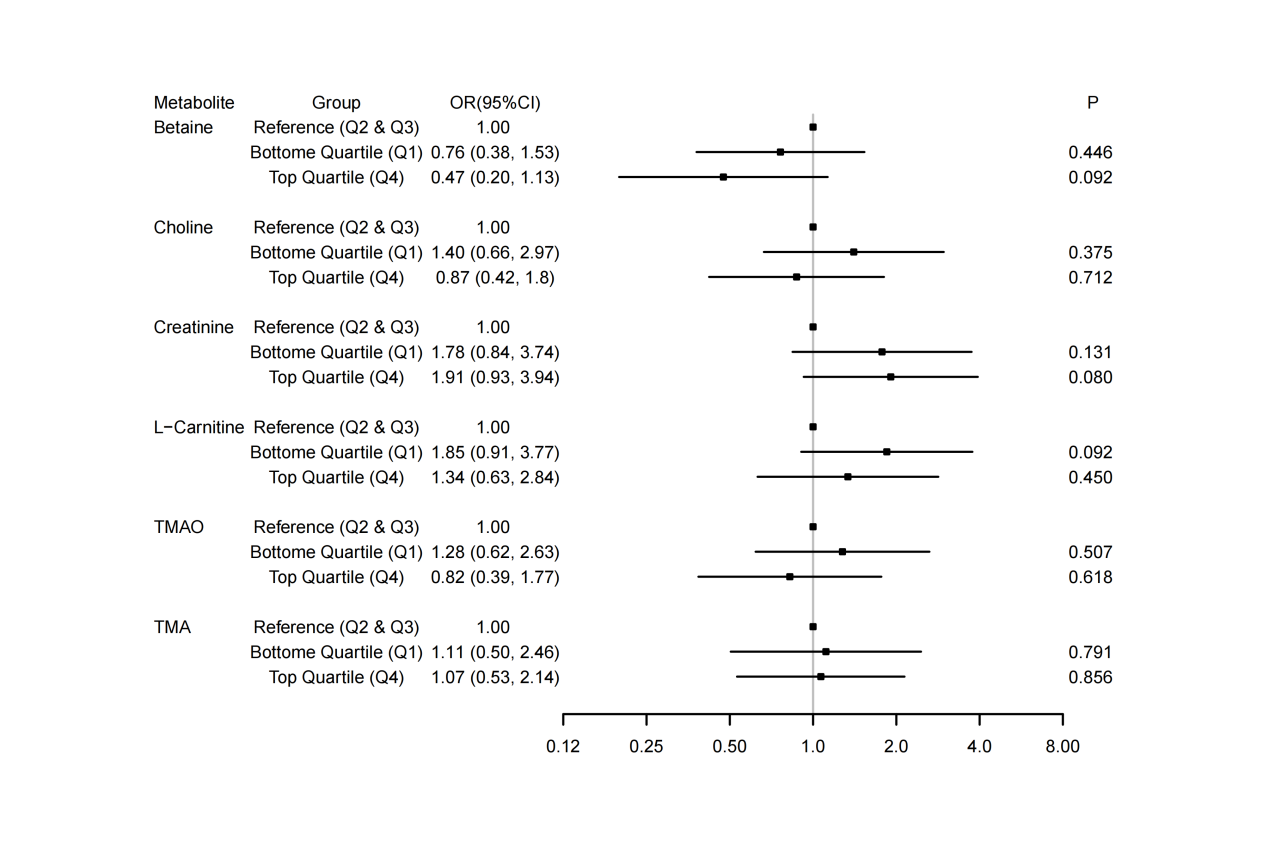


**Supplemental Figure 1.** Associations between TMAO and its precursors and the risk of abnormal fasting glucose levels. The associations were adjusted for covariates in Model 2, which included age, pre-pregnancy BMI, parity, gravidity, education, family income, and gestational weight gain.

**
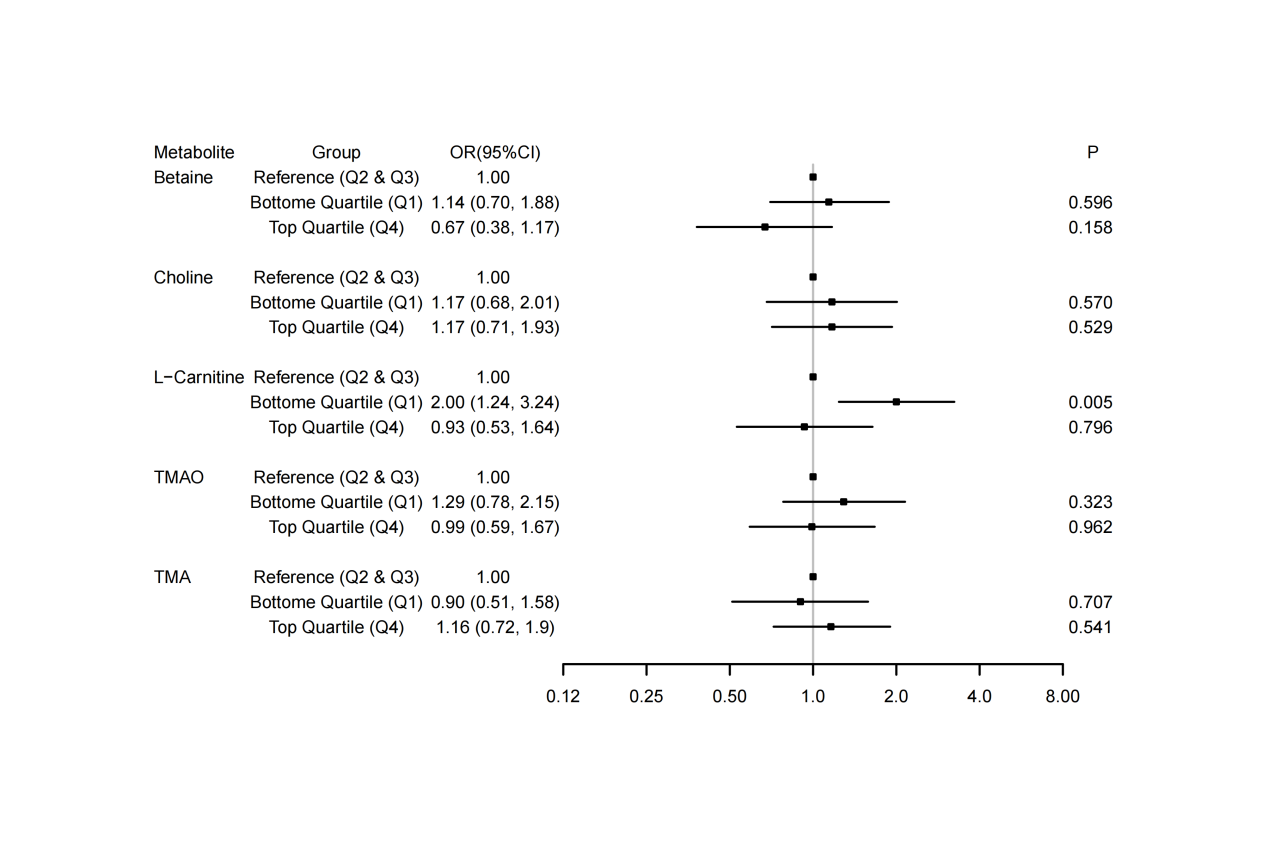
Supplemental Figure 2.** Associations between TMAO and its precursors and the risk of abnormal one-hour post-load glucose levels. The associations were adjusted for covariates in Model 2, which included age, pre-pregnancy BMI, parity, gravidity, education, family income, and gestational weight gain.


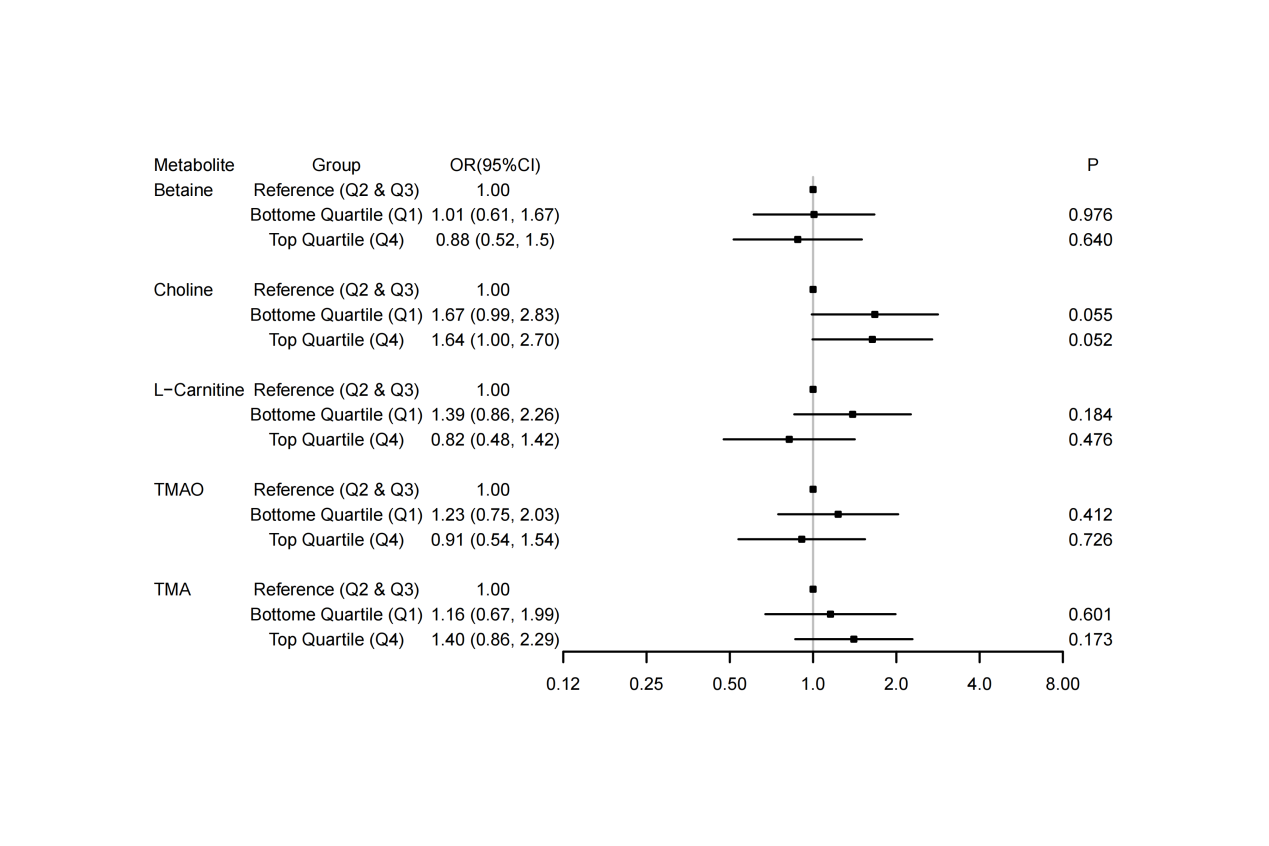


**Supplemental Figure 3.** Associations between TMAO and its precursors and the risk of abnormal two-hour post-load glucose levels. The associations were adjusted for covariates in Model 2, which included age, pre-pregnancy BMI, parity, gravidity, education, family income, and gestational weight gain.


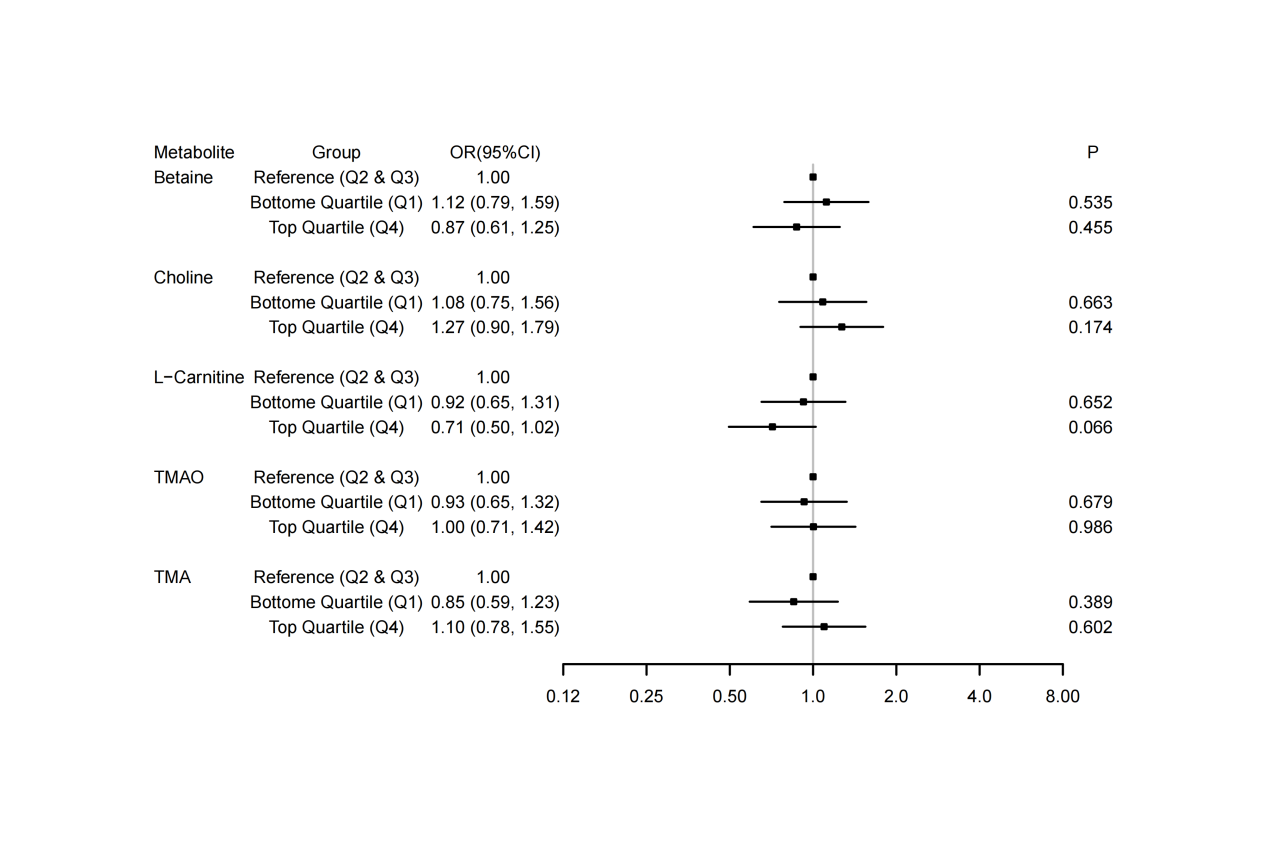


**Supplemental Figure 4.** Associations between TMAO and its precursors and the risk of one-hour post-load glucose ≥ 8.6 mmol/L levels. The associations were adjusted for covariates in Model 2, which included age, pre-pregnancy BMI, parity, gravidity, education, family income, and gestational weight gain.

**
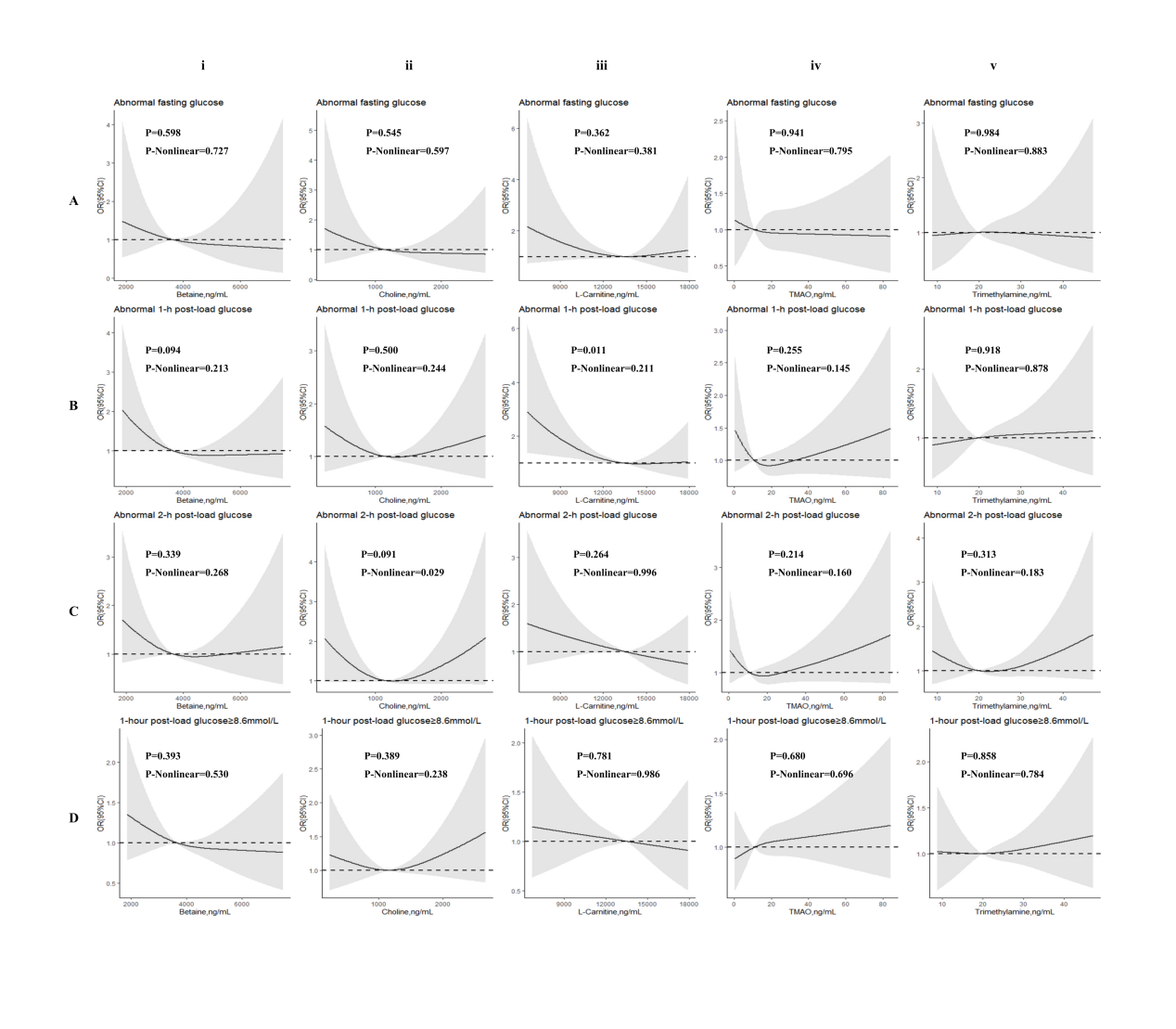
Supplemental Figure 5.** Dose-response associations between TMAO and its precursors and abnormal glucose levels. Analyses were performed by restricted cubic spine regressions (3 knots). Parts A-D represent the dose-response associations of GDM, abnormal fasting glucose, abnormal 1-hour post-load plasma glucose, abnormal 2-hour post-load plasma glucose, and 1-hour post-load plasma glucose ≥ 8.6 mmol/L, respectively. Parts i-v represent the dose-response associations of betaine, choline, L-carnitine, trimethylamine N-oxide (TMAO) and trimethylamine (TMA) , respectively.
